# Decisional Regret in Female Oncofertility Decision Making—An Integrative Narrative Review

**DOI:** 10.3390/cancers13194735

**Published:** 2021-09-22

**Authors:** Vânia Gonçalves

**Affiliations:** Centre for Health Studies and Research of the University of Coimbra (CEISUC), Faculty of Economics, University of Coimbra, Av. Dias da Silva, 165, 3004-512 Coimbra, Portugal; vmo.goncalves@hotmail.com

**Keywords:** decisional regret, female patients at reproductive age, decision-making, oncofertility

## Abstract

**Simple Summary:**

Women diagnosed with cancer at reproductive age often face potential impairments in fertility due to cancer treatments and complex and uncertain fertility decisions. The complexity of the decision-making process is often associated with psychological distress and a potential for long-term decisional regret around a decision made at diagnosis, which may have a negative impact on patients’ Quality of Life (QoL). Some factors have been associated with the experience of regret, such as patients’ perceived quality and satisfaction with the fertility counseling received, the decision to undergo fertility preservation procedures, desire for children and decisional regret. Awareness of these factors is of utmost importance to support and guide this population through their fertility decision-making process.

**Abstract:**

It is well established that fertility is an important issue for young women with cancer at reproductive age, as many have not initiated or completed their parenthood goals when diagnosed. Because cancer treatments may impair fertility, women face fertility decisions that are often complex and surrounded by uncertainty. This may put patients at risk for psychological distress and the experience of regret regarding decisions made at diagnosis, which may be associated with a negative impact on women’s QoL. This narrative review addresses current knowledge about decisional regret regarding fertility preservation decisions in adult female cancer patients at reproductive age. Electronic searches were conducted on Pubmed database for articles published in English from 1 January 2000 to 1 July 2021 that assessed decisional regret following fertility decisions in young women diagnosed at childbearing age. Of the 96 articles identified, nine provided information on decisional regret regarding fertility decisions. Studies reported that, overall, decisional regret regarding oncofertility decisions was low. Factors associated with the experience of decisional regret were patients’ perceived quality and satisfaction with fertility counseling received, the decision to undergo fertility preservation, desire for children and decisional conflict. Health providers should be aware of the factors that are potentially modifiable and prone to improvement in order to reduce decisional regret. All efforts should be made to improve availability of and access to tailored high quality fertility counseling and fertility preservation. Given the growing evidence that decision aids (DAs) are effective in increasing knowledge and reducing decisional conflict and regret, their use in a routine and timely manner to complement fertility counseling is recommended.

## 1. Introduction

The importance of fertility to young cancer patients and the wish to have biological children in the future is well established. Potential infertility due to the negative impact of a cancer diagnosis and gonadotoxic treatments on their fertility is a distressing aspect of their condition [1,2]. Chemotherapy, radiotherapy, hormonal, medical and surgical interventions may have the potential to affect female fertility. The degree to which chemotherapy and radiotherapy impact gonadal function depends on the agent administered, the dose, patient age and levels of ovarian reserve at the time. For example, women receiving bone marrow transplantation or high-dose alkylating agents for leukemia or Hodgkin’s lymphoma are at potential risk for infertility [3]. Undoubtedly, the growing development and use of fertility preservation procedures in oncology settings represent an opportunity for patients who face infertility due to cancer treatments to achieve the desired biological parenthood in the future. International and national clinical practice guidelines acknowledged the importance of including fertility as a significant part of clinical management for patients at reproductive age, advocating that health care professionals involved in young cancer patients’ care have the duty to inform and discuss with their patients the risk of infertility and fertility preservation options as early as possible and refer to fertility specialists to support patients to make well-informed decisions [4,5,6,7]. Established fertility preservation options for women, such as cryopreservation of embryos, oocytes or ovarian tissue, are available before cancer treatment begins. In addition, family building alternatives, such as adoption or third-party reproduction, often involve a complex path. In fact, it is widely accepted that women diagnosed with cancer at reproductive age often face complex fertility decisions that may place the patient and the medical team in a uniquely challenging position [8]. Oncofertility decisions usually take place within a short timeframe surrounding the time when the news of the diagnosis is delivered to the patient and other important cancer-related decisions occur, which may be understandably associated with a considerable amount of emotional turmoil for the patient. Furthermore, fertility decisions are rarely confined to only one decision, but comprise several decisions, involving multiple options with different risks, benefits and future outcomes; in addition, future difficult ethical and legal issues need also to be considered. In this context, decision-making is surrounded by uncertainty given the preference-sensitive nature of the decision, as medical evidence and clinical expertise suggest that there is more than one reasonable medical option, and the choice of what is the best option for patients depends on their preferences, characteristics and circumstances. Furthermore, despite growing developments in the field of oncofertility, there are still unanswered questions regarding many issues. For example, the likelihood of success of many fertility options is largely unknown; thus, uncertainty can also be present in this context when scientific evidence is insufficient or inconclusive to lead to definite conclusions [9,10]. Therefore, this considerable amount of uncertainty can contribute to confusion and doubt about the best course of action to be taken [9]. The ultimate fertility decision is defined by an interplay of factors, from the personal, familial, medical, ethical and spiritual levels, and may have short and long-term consequences on patients’ Quality of Life (QoL) and mental health [11].

In oncofertility, the complexity of the decision-making process, often associated with emotional consequences for the patient [12], leads, in some instances, to delays in decisions and avoidance [13]. This may cause patients to miss opportunities for future childbearing and increase the risk of experiencing difficulties associated with family-building plans. In addition, there are reports of decisional conflict and long-term distress, with a potential for long-term decisional regret around a decision made at diagnosis [14].

A better understanding of the experience of decisional regret in oncofertility will support clinical practice towards an optimal decision-making process and contribute to improved awareness regarding the needs of this patient population during their survivorship years. The aim of this narrative review is to address current knowledge about decisional regret regarding fertility preservation decisions in young adult female cancer patients at reproductive age. Factors associated with decisional regret in oncofertility decision-making are discussed. In addition, interventions to facilitate the decision-making process and reduce the probability of future decisional regret are presented. The main research question framing this review was: What is the experience of decisional regret in young women at reproductive age diagnosed with cancer?

## 2. Overview of Decisional Regret in Medical Decision-Making

Despite a lack of consensus regarding its conceptualization [15], in general, decisional regret is defined as a negative emotion involving distress or remorse following a decision [16], namely when the individual realizes or imagines that his/her current situation would be more favorable if he/she had chosen a different decision [17]. Individuals tend to focus on how the past could have been better than it was, rather than how it could have been worse. It is associated with responsibility or self-blame, which differentiate it from other negative emotions such as disappointment [17]. Therefore, theories of regret have focused on two potential sources of regret, one associated with self-recrimination around having made a bad decision and the other arising from the knowledge that another choice would have resulted in a better outcome [18,19]. Some authors argued that decisional regret does not remain static and may vary over time [15], recommending the distinction between immediate and delayed decisional regret. Decisional regret in medical contexts, namely oncology, is often measured with the decisional regret scale (DRS) that assesses regret in patients who have already made a medical decision [16]. The DRS consists of five statements: (1) it was the right decision; (2) I regret the choice that was made; (3) I would go for the same choice if I had to do it over again; (4) the choice did me a lot of harm; and (5) the decision was a wise one. Items are scored on a five-point Likert scale, ranging from 1 to 5. Items 2 and 4 are reverse coded. Higher scores indicate greater regret. This scale has established psychometric properties, being available in different languages [16].

In cancer care, research has shown that decisional regret is a common consequence of preference-sensitive decisions [20], given the context of uncertainty surrounding many decisions, especially when there is no clearly preferable clinical option. It has been associated with poorer medical outcomes, decreased QoL, lower satisfaction with decision-making and negative experiences with the health care system [16,21]. Given these possible consequences, it constitutes an indicator for assessing the quality of health decisions [15] and an important patient-reported outcome measure [21]. Several factors have been associated with the experience of decisional regret after decision-making in oncology settings. Higher levels of decisional conflict, less satisfaction with the information provided by health care providers and less involvement in the decision-making process have been significantly associated with higher decisional regret [21]. Particularly, research has demonstrated the influence of decisional conflict on decisional regret. Decisional conflict is defined as a state of uncertainty about which course of action to take when the choice among competing actions involved risk, loss, anticipated regret over the positive aspects of rejected choices or challenges to personal life values [22]. The availability of multiple paths of action that can contribute to decisional conflict may also serve to provide ready options for thoughts of “if only” that contribute to later regret [16]. Among different contributory factors for higher decisional conflict is the lack of, or deficient information about the different options under consideration and their consequences [22]. High quality evidence suggests that decision support interventions such as decision aids (DAs) and/or clinical counseling can be effective in reducing decisional conflict and also increase decision-specific knowledge and satisfaction with health care [23]. This can in turn reduce the risk of decisional regret and, therefore, improve the capability of making quality informed decisions consistent with one’s personal values [22].

## 3. Decisional Regret in Oncofertility Decision-Making

To address the research question, a narrative review was conducted by searching peer-reviewed journals published in English in PubMed database from 1 January 2000 to 1 July 2021, limiting the search to human females. The search was conducted using combinations of these keywords: “cancer AND fertility OR oncofertility OR fertility preservation AND regret”. The following inclusion criteria were applied: (i) studies including young female adult patients of childbearing age with a diagnosis of any type of cancer; (ii) primary research reporting on regret following fertility decisions after a diagnosis of cancer as an outcome variable. Given the discrepancies in the literature about the definition of young women, for the purposes of this review, “young” refers to women 45 years of age or younger at the time of diagnosis. Review articles, conference abstracts, editorials, commentaries, correspondence or case reports were not evaluated for this review. In total, 96 articles were retrieved. Of those, nine articles met the inclusion criteria and were included in the review [24,25,26,27,28,29,30,31,32]. Table 1 and Table 2 list all studies included in this review and summarize the information specific to the type of study, aims, sample, study design, decisional regret measures and relevant findings concerning decisional regret identified in each of these studies.

The extent of regret following fertility related decisions has been evaluated by studies assessing perceptions and the quality of decisions and fertility counseling [24,25,26,27,28,29] and in contexts of validation of educational instruments to assist decision-making, such as fertility-related decision aids, as an outcome measure [30,31,32]. All of these studies used the DRS [16] as a measurement tool to assess regret regarding fertility preservation treatment decisions [24,25,26,28,30,31,32] and cancer treatment choices in the context of fertility [27,29]. While most of the studies were retrospective cross-sectional studies, four studies used a prospective longitudinal design assessing newly diagnosed cancer patients in the immediate time after the decision [28,30,31,32]. Therefore, across studies, the timing of the DRS assessment varied widely, from shortly after the decision to 11.6 years subsequent to fertility decisions. With the exception of Chan et al. [27] and Campbell and Hillemeier [29], which assessed only women with gynecologic cancer and breast cancer, respectively, the majority of studies included women with mixed diagnoses in their samples, breast cancer being the most prevalent diagnosis. Table 1 summarizes the studies assessing decisional regret regarding fertility decisions, and Table 2 summarizes the studies assessing decisional regret in the context of DA validation.

### 3.1. The Experience of Regret

Collectively, the reviewed studies indicated that, in general, decisional regret was low among young women at reproductive age after facing fertility decisions following a cancer diagnosis. However, it should be noted that there is no consensus on a specific cut-off point for clinically significant decisional regret based on the DRS scores [21]. Studies reporting regret scores indicated mean scores on the DRS that ranged from 6.6 to 11 out of 25 [24,26] and a median score on the DRS of 8 out of 25 [25]. Melo et al. [28] documented mean regret scores of 1.40 for the total sample. In addition, Campbell and Hillemeier [29] reported a regret score of 1.59 for their sample, after using only four items of the DRS.

Three studies prospectively evaluating decisional regret in order to validate the effectiveness of a DA for fertility-related decisions (intervention group) in comparison with a consumer guide [30], brochures [31] or usual care [32] (control group) assessed regret at two time points following fertility decisions [30,31,32]. After baseline enrollment/assessments, regret was measured at 1 month [30,32] or 6 weeks [31], showing standardized total DRS scores ranging from 13.9 to 19.7 out of 100 for the control group, while in the intervention group scores ranged from 14.2 to 24.4 out of 100. At 6 months [31] or 12 months [30,31] DRS assessment, regret scores ranged from 17.6 to 49.1 out of 100 for the control group, while in the intervention group scores ranged from 12.94 to 45.8 out of 100. The lowest level of regret scores, at the two assessment time points, either in the control or intervention groups, were documented by Ehrbar et al. [32]. The intervention group showed reduced regret over time when compared to the control group; however, this difference did not reach statistical significance [32]. Garvelink et al. [31] reported a minor increasing trend in decisional regret over time in both groups. Peate et al. [30] found that participants in the intervention group had significantly lower decisional regret scores at 12-month assessment, after adjusting for education.

### 3.2. Factors Associated with Regret

#### 3.2.1. Fertility Counseling and the Decision to Preserve Fertility

Some studies reported on the association between the provision of fertility counseling before cancer treatment and the decision to preserve fertility and the experience of regret (Table 1). Letourneau et al. [24], in a large study of female reproductive-age survivors with mixed diagnosis, found that women who received fertility counseling from both the oncology team and the fertility specialist experienced significantly less regret about their decision to preserve fertility than women counseled only by the oncology team. Furthermore, among women who were counseled only by the oncologist, those who decided to undergo fertility preservation reported significantly less decisional regret. After adjusting for age at diagnosis, cancer type and parity at diagnosis, counseling by a fertility specialist before cancer treatment and pursuing fertility preservation remained significant predictors of decisional regret. The importance of counseling was also demonstrated in the study conducted by Chan et al. [27] with young women with early-stage gynecologic cancer. This study demonstrated that counseling provided by the oncologist or surgeon about the impact of treatments on fertility reduced the experience of decisional regret compared to the nonexistence of counseling. In addition, satisfaction with the counseling provided was associated with lower regret scores than counseling that was perceived as unsatisfactory. Additionally, undergoing fertility-sparing surgery (where the uterus and at least one ovary are retained, such as in cold knife conization, trachelectomy, unilateral salpingo-oophorectomy or ovarian cystectomy) was independently associated with reduced regret [27]. Similarly, lower decisional regret was observed in women with different cancer diagnoses (most prevalent diagnosis was breast cancer) who decided to pursue fertility preservation compared to women who decided not to preserve their fertility [26]. In the group of women who did not preserve their fertility before cancer treatment, lack of time (although, the study did not discriminate reasons for lack of time) and emotional distress were identified as factors significantly related to increased regret, while not wanting children was associated with less regret. In addition, in this group of patients, there was a trend of reduced regret when patients were counseled about fertility before cancer treatment. Following this line, Melo et al. [28] also reported that higher decisional regret regarding a fertility preservation decision was strongly associated with less decisional satisfaction. In addition, those patients that decided not to opt for fertility preservation expressed significantly higher decisional regret and lower decisional satisfaction with their fertility decisions than those who chose to pursue fertility preservation. More perceived pressure to select a specific option at the time of the decision and after cancer treatment, less perceived time available to make a decision and less certainty about the decision after cancer treatment were associated with higher regret. Similarly to Chan et al. [27], a recent study captured the importance of providing counseling that was perceived by the women as adequate and satisfactory [29]. In this regard, as perceived fertility information adequacy increased, decisional regret significantly decreased among women who received fertility counseling after finishing treatment or before and after finishing treatment. Authors concluded that the content and quality of fertility information provided during fertility counseling in addition to women’s understanding of that information may be vital for fertility counseling to have a positive effect on women’s QoL after treatment.

#### 3.2.2. Desire for Children

The desire for more children at the time of cancer diagnosis was associated with the experience of higher regret in a sample of women at reproductive age with gynecologic cancer [27].

#### 3.2.3. Decisional Conflict

Factors associated with the decision-making process, such as decisional conflict, were associated with regret. A Dutch study using a sample of 64 women with different cancer diagnoses [25] found that decisional conflict was significantly related to decisional regret. Similar results were obtained when authors analyzed a sub-sample of patients who had been counseled since 2011 and who did not attempt to conceive after fertility counseling. Similarly, a randomized controlled study evaluating the long-term effectiveness of an online DA for female patients regarding fertility preservation found a positive association between decisional conflict and decisional regret at 12-month assessment [32].

## 4. Practical Implications for Oncofertility Care

**Figure 1:**
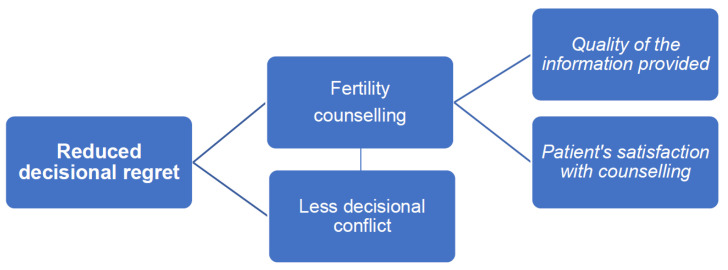
Potentially modifiable factors in clinical practice associated with reduced fertility decisional regret.

According to the studies focusing on decisional regret in oncofertility decision-making, factors related to the decision-making process such as less decisional conflict, the existence of fertility counseling and the quality of the information provided in addition to patients’ satisfaction with the counseling received were significantly associated with women’s experience of reduced regret about their fertility decisions. This provides essential information to health care professionals involved in the care of women at reproductive age with cancer, as these factors are potentially modifiable and prone to improvement in order to help prevent, reduce or manage decisional regret (Figure 1). Unfortunately, there are other factors related with decisional regret that may be unavoidable, such as, for example, when women desire children at diagnosis and face potential infertility due to cancer treatments. For those patients, psychological support, integrated into a multidisciplinary approach to oncofertility care, should be provided within the immediate impact of their diagnosis and complex fertility decision-making process and also during long-term survivorship, taking into consideration that fertility attitudes and psychosocial adjustment may change over time [33,34].

Fertility and fertility preservation are important issues to be considered during cancer management for patients at reproductive age. The possibility of undergoing fertility preservation procedures after potential infertility due to cancer treatments may offer women different rewards, such as, for example, the hope of bearing a child in the future that may represent, for some patients, a sense of normalcy and fulfilling life [2]. As documented by some studies addressing decisional regret, women who decided to preserve their fertility experienced less decisional regret with their fertility decisions. However, to reach an informed, high-quality fertility decision, it is crucial to provide tailored fertility counseling and support during such an uncertain and complex decision-making process.

Overall, the decision-making literature in medical settings corroborates the view that the provision of comprehensive information by health care professionals that is relevant to patients’ individual needs is an essential component of a decision-making process [35]. Given the high level of uncertainty and complexity involved in fertility decisions, a high-quality decision about using fertility preservation requires that patients be fully informed about the diverse fertility options being offered to them, realistic expectations and potential fertility risks involved [2]. Furthermore, it is important that patients perceive that their personal values and preferences have been taken into account and are satisfied with the decision-making [36]. Fertility counseling is also important for women at reproductive age that are not candidates for fertility preservation options [24], since it may provide them an opportunity and context to grieve and adapt psychologically. The grief process is facilitated by shared decision-making between the patient and the clinician through an open dialogue on risks, benefits, options and the reality of the patient’s situation. When appropriately counseled about fertility preservation, cancer patients report also less decisional conflict [14], which is positively associated with future experiences of decisional regret, as demonstrated by the studies presented. This may have a subsequent impact on patients’ experience of a better quality of life post cancer treatment. Therefore, the psychological impact of loss or impaired fertility may lessen with access to appropriate and timely oncofertility care, which may influence the experience of infertility for cancer patients [37]. However, contrary to recommendations advocated by several national and international clinical guidelines about the important role played by health providers in supporting the efforts of patients to reach a high-quality, informed decision, it is widely acknowledged in the literature that some cancer patients do not receive timely and adequate fertility counseling prior to their cancer treatment, leaving their needs regarding fertility information often unmet [38,39,40]. This practice also conflicts with reports that stated that young cancer patients consider the provision of fertility-related information a priority [41]. Lack of fertility counseling and patients’ perceptions of dissatisfaction with the quality of the counseling received may be risk factors for patients’ future experiences of regret with their fertility decisions. From a psychological point of view, collaboration between oncology and fertility specialists seems to be linked to better decision-making and reduced regret with fertility decisions. Decisional regret studies in oncofertility have emphasized the important role of fertility specialists during fertility counseling in reducing regret and improving psychological health. This may be due to their greater fertility expertise and training that may equip them with better skills to provide fertility information and respond appropriately to patients’ reproductive concerns [42]. This review supports the improvement and optimization of a multidisciplinary collaborative model of care, grounded in a robust collaboration among different specialties, to guide oncofertility management for female cancer patients in order to improve women’s perceived low satisfaction with fertility counseling provided by their health professionals [27] and improve mental health outcomes as they navigate through their cancer trajectory. This should offer clear pathways for timely referral to fertility specialists and psychological care, in order to aid patients at the time of diagnosis and survivorship.

**Figure 2:**
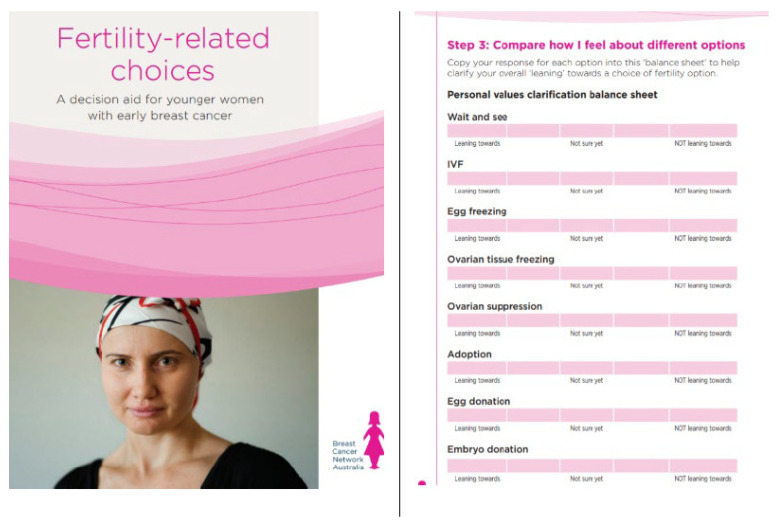
Fertility-related choices: A decision aid for younger women with early breast cancer. Permission from Dr. Michelle Peate, The University of Melbourne.

There is growing evidence for the use of educational resources to assist with fertility preservation decision-making, with DAs being regarded as a useful component of fertility counseling that increases fertility information satisfaction and knowledge and has the potential to lower decisional conflict and regret, thus helping patents to make better informed decisions [43]. DAs are educational tools designed to aid decision-making by addressing individual values and preferences [41], being particularly helpful in situations when there is limited time to make the decision, such as the case with fertility decisions [44]. DAs help make the decision explicit, describe options available, and assist patients’ understanding of options as well as their possible benefits and harms. DAs help patients to consider options from a personal perspective, contributing to a shared decision-making between patients and providers [23]. Validated educational tools to support fertility decision-making are still scarce [45]. Evaluated DAs for female fertility preservation decision-making include a DA in English for young women with breast cancer [41] (Figure 2) and another one in Dutch [31]. Recently, there was a validation for a DA for young female cancer patients diagnosed with different types of cancer in Germany [32]. This DA was already translated into French and its content adapted to three different countries, namely Switzerland, Austria and Germany.

## 5. Conclusions

Despite the methodological heterogeneity across studies focusing on decisional regret following oncofertility decisions, findings revealed that a majority of women reported low levels of decisional regret regarding fertility decisions after a cancer diagnosis. However, a subset of women may be at risk of experiencing future regret that may compromise their long-term QoL. Current available literature suggests the need to carefully identify patients at risk for psychosocial distress, difficulties in coping with fertility decision-making and reduced QoL due to fertility loss or threat of infertility related to a cancer diagnosis and treatments. It is well-documented that fertility is an integral component of women’s QoL, and all efforts should be made to improve availability of and access to high-quality fertility counseling and fertility preservation. The quality of the fertility counseling provided correlates positively with better decision-making outcomes, such as decisional conflict and regret. Given the growing evidence that DAs are effective in increasing knowledge and reducing decisional conflict and regret, and the fact that patients value educational tools to inform their choices about fertility preservation, it is recommended that they are used routinely and in a timely manner to complement fertility counseling, to assist patients in their decision-making and to support clinicians and other health professionals in their daily practice. In addition, oncofertility knowledge and communication skills training must be compulsory for the clinical multidisciplinary teams that are involved in the care of young patients. This training should equip the teams with the confidence and knowledge of how to implement high standard oncofertility care and how to engage patients effectively in fertility counseling via shared decision-making.

Given the currently limited body of research on this topic, more prospective longitudinal studies are needed to understand long-term regret following oncofertility decisions and its impact on psychological adaptation to cancer and QoL, as there is some evidence suggesting that regret may fluctuate over time. Furthermore, each cancer type brings unique clinical and psychological challenges to patients related to their decision-making experience, such as, for example, the experience of regret; therefore, it is important to understand specific needs related to a particular diagnosis in order to devise support strategies that are meaningful to these patients. In this context, qualitative studies could also provide meaningful insights regarding women’s experiences of regret and QoL following fertility decisions. Decisional regret is a complex emotion, and more knowledge needs to be gathered regarding patients’ perceptions of decision-making outcomes and consequences and their impact on patients’ adaptation to the different challenges of the cancer trajectory. Finally, this review supports the need for the development and validation of interventions designed to improve patient-provider communication and knowledge in oncofertility that may have a positive effect on the quality of decision-making and clinical practice.

## Figures and Tables

**Table 1:** Studies addressing decisional regret regarding fertility decisions of women at reproductive age diagnosed with cancer.

Study	Origin	Type	Aims	Sample	Study Design	Decisional Regret Measures	Relevant Findings about Decisional Regret
Letoruneau et al. [24]	USA	Quantitative	- To evaluate whether receiving pre-cancer treatment infertility counseling from an oncology team is associated with improved post-treatment QOL. - To evaluate whether seeing a fertility doctor or taking action to preserve fertility is associated with even greater improvements in QOL than only receiving counseling from the oncology team.	- N = 918 - Mixed diagnosis (Hodgkin’s lymphoma most prevalent—N = 286) - Mean age at dx: 31.5 - Mean age at survey: 40.9 - Mean length time since diagnosis: 9.8 years - 52% had children before TX - 54% desired children after TX	Cross-sectional	DRS—to measure the decision to undergo (or not) FP	- Mean (SD) decision regret score (women received counseling only from oncologist): 11 (5) - Mean (SD) decision regret score (women who receive counseling from oncologist + fertility specialist): 8.4 (4.4) - Receiving counseling from a fertility specialist and FP appears to decrease regret. - Women counseled about fertility by both an oncology team and a fertility specialist had significantly less regret about their FP decision than those counseled only by an oncology team - Among those women who were counseled by their oncologist, the largest difference in regret was noted between women who took action to preserve their FP and those who did not. These differences remained significant after adjustment for age at diagnosis, cancer type, and parity at diagnosis.
Basting et al. [25]	The Netherlands	Quantitative	- To investigate how female patients experienced FP consultation and FP decision-making. - To investigate the interplay between patients’ FP consultation experiences, decisional conflict and decision regret.	- N = 64 - Mixed diagnosis (Breast cancer most prevalent—60%) - Mean age: 28.9 - Mean age at survey: 40.9 - Follow-up mean: 2 years - 90% had a partner - 15% had children	Cross-sectional	DRS—to measure past FP decisions	- Median score on the decision regret scale: 8 - Decisional conflict was significantly related to decisional regret. Women who recalled decisional conflict at the time of diagnosis were significantly more likely to have current decisional regret. Similar results were obtained in a sub-sample of patients who were counseled since 2011 and who did not attempt to conceive after fertility counseling.
Benedict et al. [26]	USA	Quantitative	- To evaluate the decisions young adult female cancer survivors made about FP before treatment - To understand the extend of decision regret related to FP after TX - To compare characteristics of patients who preserved their fertility to those who did not - To identify factors related to increased regret among survivors.	- N = 159 - Mean age at TX: 33 - at least 1 year from TX (56%) - Mixed diagnosis (Breast cancer most prevalent—17%) - 81% had partner - 41% had at least 1 child before TX - 62% wanted children in future	Cross-sectional	DRS—to measure the decision to undergo (or not undergo) FP before treatment	- Average decision regret score: 10, low regret overall (SD = 4.4; median = 10; range 5–25) - Women who preserved their fertility had lower regret scores compared to those who did not. - Decisional regret was not related to age at diagnosis or current age, race or ethnicity, partner status, prior children, treatment type and time since TX. - Among women who did not undergo FP: 61% felt they made the right decision; 26% regretted their choice; 19% would not make the same choice again. - Among women who pursued FP: 84% felt they made the right decision; 10% regretted their choice; 6% would not make the same choice again. - Decision regret among those who did not undergo FP: Greater for those who expressed lack of time and emotional distress as reasons for not pursuing PF compared to women who did not report these reasons; not wanting children was related to less regret; pre-fertility counseling was associated with less regret at a trend level.
Chan et al. [27]	USA	Quantitative	- To compare regret in GYN cancer survivors who did and did not recall pre-TX fertility counseling - Secondary aim to evaluate the effect of FSS on regret and to characterize patients at highest risk of regret	- N = 470 - 228 (48.5%) cervical, 125 (26.6%) ovarian, 117 (24.9%) endometrial - Mean age at dx: 33.7 - Mean age at survey: 45.2 - 324 (69%) had children before TX - 235 (50%) desired children after TX	Cross-sectional	DRS—to measure regret following cancer treatment	- After adjusting for age at time of DX and at time of survey, counseling (*p* = 0.02) and FSS (*p* = 0.03) were associated with lower regret scores - Desire for more children at time of DX was associated with higher regret (*p* < 0.001 adjusted)
Melo et al. [28]	Portugal	Quantitative	- To assess female cancer patients’ perceptions of FP decision-making - To examine the effects of clinicians’ support on decision quality.	- N = 71 - Mean age at TX: 31.42 - Mixed diagnosis (Breast cancer most prevalent—74.6%) - 75.7% had partner - 19.7% had at least 1 child before TX	Prospective, longitudinal (T1: when participants required to make fertility decision; T2: End of TX)	DRS—to measure current regret about fertility decisions	- Mean (SD) decision regret score: 1.40 (0.59), Min: 1.00, max: 3.60 - At T2, low decisional regret and high decisional satisfaction. - Higher decisional regret about FP decision was strongly associated with less decisional satisfaction. - Participants who decided not to pursue FP had higher regret at T2 and lower decisional satisfaction than those who decided to undergo FP. - Higher regret was moderately associated with more perceived pressure to select a specific option at T1 and T2 and with less perceived time available to make a decision at T2, and regret was strongly associated with less certainty about the decision at T2
Campbel and Hillemeier [29]	USA	Quantitative	- To examine whether fertility counseling provided to pre-menopausal breast cancer patients is associated with decreased decisional regret post-treatment and whether the effects of fertility counseling receipt are influenced by information adequacy.	- N = 128 breast cancer - Mean age at diagnosis: 32.14 - Mean age at study: 37.69 - 70.19% were married	Cross-sectional	DRS—to measure breast cancer treatment choices regarding the effects they had on survival and fertility (4 items used)	- Mean (SD) decision regret score: 1.59 (0.07) - Women who received fertility counseling had a higher regret score than women who did not received counseling (difference marginally significant: *p* = 0.07) - Fertility counseling was not directly associated with decisional regret. - Fertility information adequacy was significantly associated with the relationship between fertility counseling and regret. Regret scores were significantly reduced when women receive more adequate fertility information after finishing treatment or before and after finishing treatment compared to women receiving less adequate information.

Abbreviations: TX, cancer treatment; DX, cancer diagnosis; GYN, gynecologic cancer; FP, fertility preservation; FSS, fertility-sparing surgery; QoL, quality of life; DRS, decisional regret scale; DA, decision aid.

**Table 2:** Studies addressing decisional regret in context of decision aids validation.

Study	Origin	Type	Aims	Sample	Study Design	Decisional Regret Measures	Relevant Findings about Decisional Regret
Peate et al. [30]	Australia	Quantitative	- To evaluate the efficacy of a DA compared with usual care among young women diagnosed with early breast cancer. - Specific aims: (i) Compare changes in decision-related outcomes, including decisional conflict about fertility treatment decisions and knowledge, over time. (ii) Compare decision-related outcomes, including decisional regret about treatment decisions, and informed choice at 1- and 12-months post diagnosis. (iii) Examine potential changes in anxiety and depression as a result of the use of the DA compared to usual care.	- N = 120 (Intervention group: 48; Control group: 72) - Newly diagnosed early-stage breast cancer patients - Mean age control group: 33.8 - Mean age intervention group: 32.3 - 71.6% (control group) and 76.6% (intervention group) had - 36.1% (control group) and 25% (intervention group) had children	Prospective study Non-randomized trial design Control group: consumer guide; Intervention group: received the DA Baseline (1st consultation, T1 (1 month after), T2 (at 12 months)	DRS- to measure regret related to FP treatment decisions (measured T1 e T2)	-At 1 month, regret scores regarding fertility-related decisions were not significantly different between the control and intervention groups. -After adjusting for education, the intervention group had significantly lower decisional regret at 12 months.
Garvelink et al. [31]	The Netherlands	Quantitative	-To test the feasibility and effects of the detailed DA compared to brochures about FP on decisional conflict, knowledge, regret, and reproductive concerns.	- N = 36 (Intervention group: 13; Control group: 13) - Newly diagnosed breast cancer patients - Mean age control group: 32.9 - Mean age intervention group: 35.8 - 92% (in control and (intervention group) had male partner - 46% (in control and (intervention group) had children	RCT Control group: Informational brochures Intervention group: received the DA Baseline (T0), T1 (6 weeks after T0), T2 (6 months after T0)	At T1 and T2: DRS- measure regret related to FP (measured T1 e T2)	- Both groups showed a trend for a minor increase in regret between T1 and T2.
Ehrbar et al. [32]	Switzerland	Quantitative	- Secondary analysis of the results of an RCT evaluating a DA for female patients with different cancer diagnoses: - To address the long-term impact of DA on knowledge and attitude - To explore its long-term effectiveness regarding decisional regret - To investigate the association between decisional conflict and decisional regret over time.	- N = 51 (Intervention group: 27; Control group: 24) - Different diagnosis, majority breast cancer - Mean age control group: 28.78 - Mean age intervention group: 29.92 - 85.2% (control group) and 75% (intervention group) had - 14.8% (control group) and 12.5% (intervention group) had children	RCT Control group: usual care Intervention group: received the DA T1: directly after fertility counseling; T2: 1 month later; T3: 12 months later.	DRS- to measure regret related to FP treatment decisions (measured T2 e T3)	- Decisional regret low and stable in all participants. - Decisional regret was lower in the intervention group when compared to the control group, but the difference was not significant. - Positive association between decisional conflict and decisional regret at T3 (12 months).

Abbreviations: TX, cancer treatment; DX, cancer diagnosis; GYN, gynecologic cancer; FP, fertility preservation; FSS, fertility-sparing surgery; QoL, quality of life; DRS, decisional regret scale; DA, decision aid; RCT, randomized controlled trial.
